# Potentiating NK cell activity by combination of Rosuvastatin and Difluoromethylornithine for effective chemopreventive efficacy against Colon Cancer

**DOI:** 10.1038/srep37046

**Published:** 2016-11-14

**Authors:** Naveena B. Janakiram, Altaf Mohammed, Taylor Bryant, Yuting Zhang, Misty Brewer, Ashley Duff, Laura Biddick, Anil Singh, Stan Lightfoot, Vernon E Steele, Chinthalapally V. Rao

**Affiliations:** 1 Center for Cancer Prevention and Drug Development, Department of Medicine, Hematology Oncology Section, Stephenson Cancer Center, University of Oklahoma Health Sciences Center, Oklahoma City, OK 73104, USA; 2 Chemopreventive Agent Development Research Group, Division of Cancer Prevention, National Cancer Institute, Bethesda, Maryland, USA.

## Abstract

Colorectal cancer (CRC) is the second highest cause of cancer-related deaths. A successful strategy to improve chemopreventive efficacies is by down-regulating tumor polyamines and enhancing NK cell activities. Colonic carcinogenesis was induced by azoxymethane (AOM) in male F344 rats. Eight weeks after AOM treatment, animals were fed diets containing Rosuvastatin and difluromethylornithine (DFMO) individually and in combination for 40 weeks. Both agents showed significant suppression of adenocarcinoma multiplicity and incidence with no toxicity compared to untreated rats. Low-dose Rosuvastatin plus DFMO suppressed colon adenocarcinoma multiplicity by 76% compared to low-dose Rosuvastatin (29%) and DFMO (46%), suggesting additive efficacy. Furthermore, low-dose combination caused a delay in colonic adenocarcinoma progression. DFMO, Rosuvastatin and/or combinations significantly decreased polyamine content and increased intra-tumoral NK cells expressing perforin plus IFN-γ compared to untreated colon tumors. Further *ex-vivo* analysis of splenic NK cells exposed to DFMO, Rosuvastatin or combination resulted in an increase of NKs with perforin expression. This is the first report on Rosuvastatin alone or combination strategy using clinically relevant statin plus DFMO doses which shows a significant suppression of colon adenocarcinomas, and their potential in increasing functional NK cells. This strategy has potential for further testing in high risk individuals for colon cancer.

Colorectal cancer is the second leading cause of cancer deaths. In 2015, about 145,000 new cases of colon cancer and 48,000 deaths were reported^1^. Though colon cancer is detectable and curable by surgery, deaths are mostly due to recurrence following surgery. It is observed in literature that about 20–40% colon cancer survivors develop high risk adenomas^2^,^3^. As per epidemiological, clinical and preclinical data, NSAIDs are said to be the most effective drugs in treating or preventing adenomas and colorectal cancers (CRC) but their use results in stomach, intestinal bleeding and serious cardiovascular effects^4^. So better alternatives, such as low dose combinations of well-established non-toxic agents are to be developed for the prevention of CRC.

Statins are lipid lowering drugs used by large populations due to their safety and effects on reducing cardiovascular disease and reducing deaths^5^. Statins, apart from their lipid lowering ability, also have various other effects such as modulation of cell growth, inhibition of inflammation, and induction of apoptosis^6^. Many reports suggest that statins have potential chemopreventive properties in CRC^7^,^8^. Experimental evidence by us and other labs demonstrated that statins have been effective in both *in-vitro* human colon cancer cells and in *in-vivo* animal studies, such as in xenografts, genetically predisposed animal model, and in carcinogen induced CRC models, individually or in combination with COX inhibitors^9^,^10^,^11^,^12^,^13^,^14^. Previous small prospective studies reported weak/no significant reduction in risk for CRC with statin use with any dose levels^15^. Whereas, the Molecular Epidemiology of Colorectal Cancer (MECC) study, a very large study with 4,000 people, reported 45% risk reduction in CRC with 5 years of statin use and a retrospective cohort study reported 35% reduction in US veterans^16^,^17^. A recent cohort study with CRC patients, reported statin use after CRC diagnosis improved the survival of patients^18^. As the outcomes from clinical studies are conflicting with use of statins alone because of retrospective observatory designs, combining with other chemopreventive agents may provide an efficacy benefit in CRC prevention.

It is well documented that endogenous and exogenous sources of polyamines have a significant impact on growing tumors. D,L-α-difluoromethylornithine (DFMO) is an irreversible inhibitor of ornithine decarboxylase first enzyme in synthesis of polyamines^19^ and has been shown to be an effective chemopreventive agent for many organ site cancers^20^,^21^,^22^,^23^. Polyamines are reported to act as natural immune suppressors by decreasing cytolytic properties of NK cells, thus protecting the tumor from the host’s immune responses. Polyamine deprivation stimulates NK cell activity. On the basis of reported studies, statins may directly inhibit the cholesterol pathway metabolites leading to the inhibition of activation K-ras/RhoB proteins. In addition, statins may limit exogenous polyamine intake by blocking caveolae or membrane pits^24^. Statins are less investigated for their immune modulating capabilities in CRC. We show here that low-dose combination DFMO and Rosuvastatin provide additive chemopreventive efficacy and significantly enhance innate immune cells like NKs in colonic tumors.

## Results

### General Health Observations

**Figure 1:**
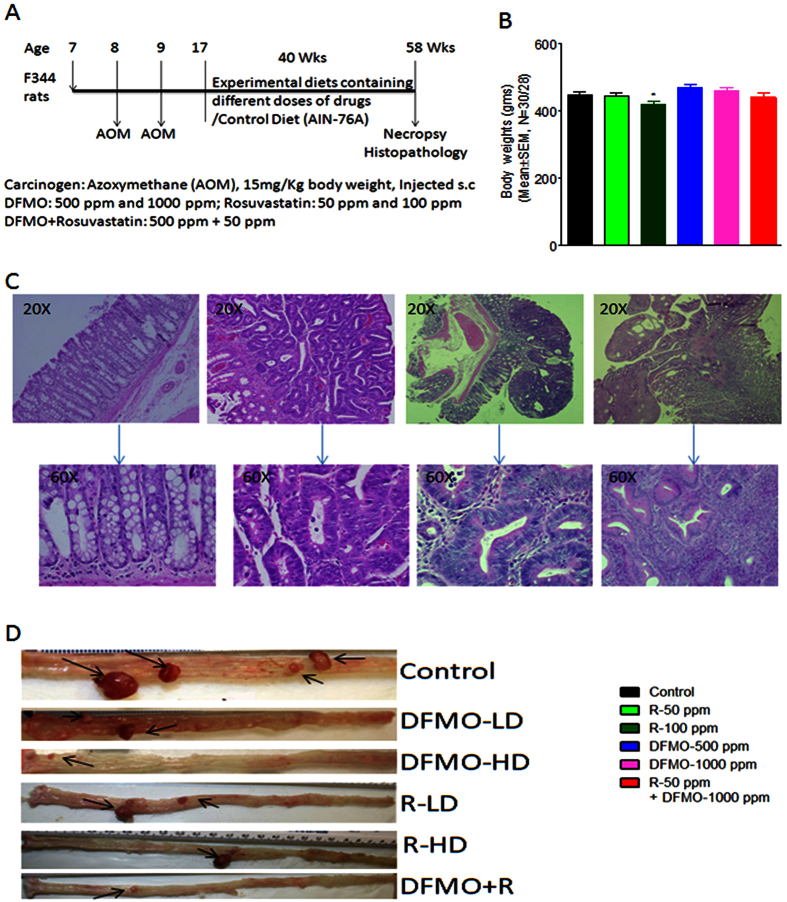
(**A**) Experimental design for the evaluation of the chemopreventive efficacy of DFMO, Rosuvastatin, and combination of DFMO plus Rosuvastatin on AOM-induced CRC using F344 rats. Groups (30 rats per group) of rats were fed diets containing 0, 500, or 1000 ppm DFMO/ 50 and 100 ppm of Rosuvastatin/50 ppm Rosuvastatin plus 500 ppm of DFMO from 17 weeks of age to the end of the experiment. The study was terminated after 40 weeks of exposure to the experimental diets (see Materials and Methods for more details). (**B**) Final body weights of rats fed control diets and/or experimental diets containing 500 or 1000 ppm DFMO, 50 or 100 ppm Rosuvastatin and 50 ppm Rosuvastatin plus 500 ppm DFMO. No significant change in body wt was observed between drug-treated and control groups. Data are presented as means ± SEM. (**C**) Hematoxylin and Eosin staining pictures of rat AOM-induced adenoma, adenocarcinoma (**D**) Representative pictures of control, DFMO, Rosuvastatin, DFMO plus Rosuvastatin animal colons.

Upon gross examination, the tissues appeared to be normal in all the treated groups. Body weights of all animals fed the experimental diets were comparable to those of control fed diet animals (Fig. 1B). Chronic exposure of DFMO and Rosuvastatin were safe and did not produce any gross changes in liver, kidneys, lungs, and lack in overt-toxicities.

### DFMO, Rosuvastatin and combinations reduced Adenocarcinomas multiplicity and incidence

Vehicle-only treated animals did not show any tumors. As per histological grading the colon tumors were divided into adenomas, non-invasive and invasive adenocarcinomas (Fig. 1C). DFMO, Rosuvastatin individually and low dose combination treatments with these agents reduced colon tumor formations in F344 rats compared to untreated rats (Fig. 1D).

#### Total Colon adenocarcinoma multiplicity

**Figure 2:**
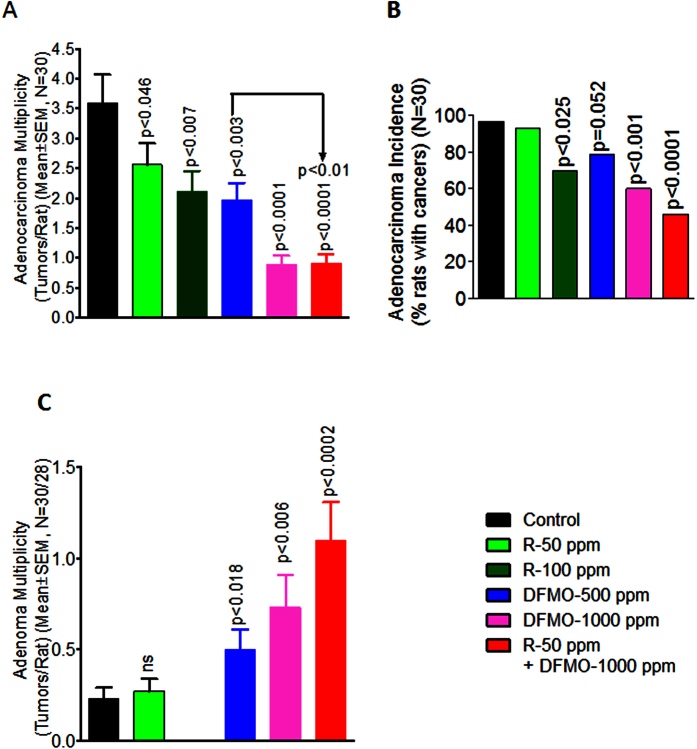
Chemopreventive effects of DFMO, Rosuvastatin, or combination of low-dose DFMO plus Rosuvastatin on (**A**) azoxymethane (AOM)-induced colon Adenocarcinoma multiplicity, (**B**) Adenocarcinoma incidence, and (**C**) Adenoma Multiplicity in male F344 rats. Data values are means ±SEM of 30 animals per treatment group with Welch’s correction. Control and treated groups are significantly different from one another (P < 0.05).

Dietary administration of DFMO showed significant dose-dependent decrease in colon adenocarcinoma multiplicity: at 500 ppm, 1.96 ± 0.29 (45.4%, P < 0.003); at 1000 ppm, 2.12 ± 0.33 (75.2%, P < 0.0001; Fig. 2A) compared to control diet fed rats (3.59 ± 0.48). A significant dose-dependent decrease in colon adenocarcinoma multiplicity by dietary administration of Rosuvastatin was observed, where Rosuvastatin at 50 ppm, showed 2.56 ± 0.36 (28.7%, P < 0.046) and at 100 ppm showed 2.12 ± 0.33 (40.9%, P < 0.007) colon adenocarcinoma multiplicity compared to control diet fed rats (Fig. 2A). Importantly, low dose combination of DFMO and Rosuvastatin showed a significant decrease in colon adenocarcinoma multiplicity 0.90 ± 0.16 (74.9%, p < 0.0001) compared to control diet fed rats. In addition, combinational low-dose showed more efficacies in inhibition adenocarcinoma as compared to individual low doses of each agent as shown in Fig. 2A.

#### Total Colon adenocarcinoma Incidence

The number of rats with colon adenocarcinomas was significantly reduced by 40% (p < 0.001) in high dose dietary DFMO treatments (Fig. 2B) compared to untreated rats. Though inhibition of colon adenocarcinoma incidence by 20.7% (p = 0.052) was observed in low dose dietary administration of DFMO compared to control diet fed rats, it did not reach statistical significance (Fig. 2B). A similar result was observed with Rosuvastatin treatments. Low dose Rosuvastatin treatment did not show statistical significance in inhibiting colon adenocarcinoma incidence, whereas 100 ppm Rosuvastatin significantly inhibited formation of colon adenocarcinoma incidence by 26.7%, p < 0.025 compared to untreated rats. Importantly, low dose combination treatment with DFMO and Rosuvastatin significantly inhibited colon adenocarcinoma incidence by 53.4% (p < 0.025) compared to untreated rats (Fig. 2B).

#### Inhibition of adenoma progression to adenocarcinoma

DFMO low dose treatment restricted adenoma progression to adenocarcinoma which resulted in a non-significant increase in adenomas in DFMO low dose treatment groups. As expected, high dose DFMO completely suppressed adenoma multiplicity (Fig. 2C). A significant dose-dependent increase in the number of adenoma multiplicity was shown by Rosuvastatin and low dose combination of DFMO plus Rosuvastatin treatments in F344 rats (Fig. 2C). Low dose combination of DFMO plus Rosuvastatin showed significant inhibition of adenocarcinomas with an increase in adenomas formation, suggesting combination drug effects on the progression delay of adenoma to adenocarcinoma formation (Fig. 2A–C).

### DFMO, Rosuvastatin and its combination enhanced the expression of Nk1.1 receptors of NK cells and their perforin and IFN-γ production in colon tumors

**Figure 3:**
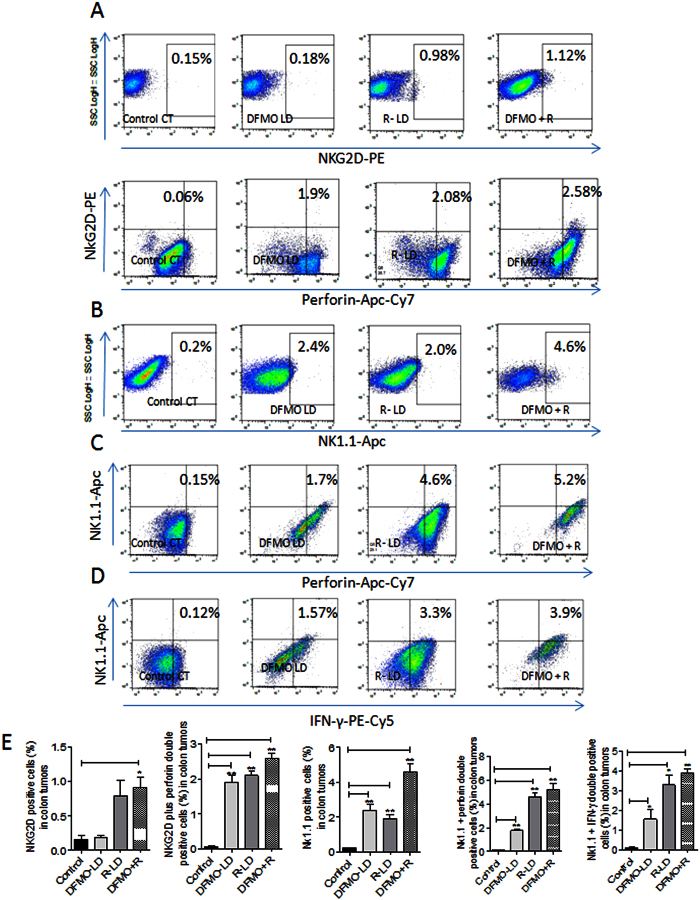
Flowcytometry analysis of functional NK cells by staining of NKG2D, Nk1.1, perforin and IFN-γ in control and treated colon tumors (**A**) The colon tumor cells are gated on lymphocytes, and analyzed for NKG2D positive cells (upper panel) and the colon tumor cells are analyzed for cells that are double-positive for NKG2D and perforin. The dot plot shows the double-positive cells at the left hand corner of each plot (lower panel). (**B**) The colon tumor cells are gated on lymphocytes and analyzed for cells that are positive for NK1.1. (**C**) The colon tumor cells are gated on lymphocytes and analyzed for cells that are double-positive for NK1.1 and perforin. The dot plot shows the double-positive cells at the left hand corner of each plot. (**D**) The colon tumor cells are gated on lymphocytes and analyzed for cells that are double-positive for NK1.1 and IFN-γ. The dot plot shows the double-positive cells at the left hand corner of each plot. (**E**) The bar graphs shows the percentages of cells positive for NKG2D, NKG2D plus perforin, NK1.1, NK1.1 plus perforin and NK1.1 plus IFN-γ in control and treated colon tumors. The flowcytometry analyses of different experimental group animals were compensated with unstained, positive/isotope controls. The numbers indicate the percentage of the gated cells out of the total number of cells within the plot.

Functionally active NK cells were analyzed using NK cell receptor markers NK1.1, NKG2D, perforin and Interferon-γ (IFN-γ) in treated and untreated colon tumors (Fig. 3). There was a significant decrease in perforin and Interferon-γ-expressing NK cells in control colon tumors. Low doses of DFMO and Rosuvastatin alone showed a non-significant increase in NKG2D receptor expressing NK cells. However, low dose combinations of these drugs significantly increased the expression of NKG2D receptor on NK cells. In comparison to low doses of DFMO and Rosuvastatin alone treatments, the combination treatments of these drugs increased the expressions of perforin and IFN-γ in NKG2D receptor positive NK cells in colon tumors (Fig. 3).

Similarly, DFMO and Rosuvastatin-alone treated colon tumors showed increased NK1.1 receptor expressions compared to control untreated colon tumors (Fig. 3). The combination of DFMO and Rosuvastatin in colon tumors significantly increased NK1.1 receptor positive cells compared to individual low doses of these agents alone and control colon tumors (Fig. 3). DFMO and Rosuvastatin-alone treated colon tumors showed NK1.1 receptor positive NK cells expressing increased perforin and IFN-γ compared to NK1.1 receptor positive cells in control colon tumors. Whereas, low dose combination of DFMO and Rosuvastatin significantly increased NK1.1 receptor positive NK cells with perforin and IFN-γ expressions compared to individual low doses of these agents and control tumors (Fig. 3).

### Polyamine inhibited expression of NK1.1 receptors of NK cells and their perforin and IFN-γ production, is reversed by DFMO, Rosuvastatin and its combination

**Figure 4:**
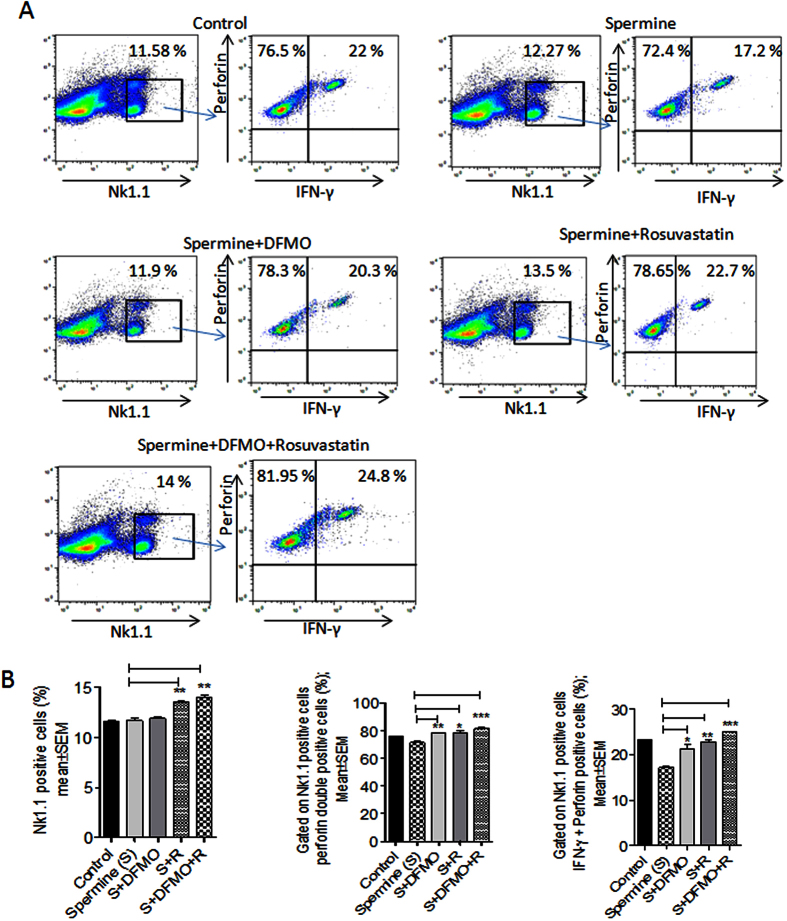
DFMO plus Rosuvastatin combination increase NK cell function (**A**) The splenic cells were gated on NK1.1 and analyzed for cells that are positive for perforin and IFN-γ with different treatment cell samples (**B**) The bar graphs shows the percentages of cells positive for NK1.1, NK1.1 plus perforin, and NK1.1 plus IFN-γ in control and treated splenic cells. The numbers indicate the percentage of the gated cells out of the total number of cells within the plot.

As the treated colon tumor samples showed increase in NK cells expressing perforin and IFN- γ, we further conducted an *ex-vivo* study using DFMO and Rosuvastatin on isolated NK cells from mice splenocytes to observe if the drugs can induce any phenotypic and functional alterations of NK cells. Spermidine treated NK cells showed a decrease in NK cells expressing perforin and IFN-γ, whereas when NK cells were treated with DFMO and Rosuvastatin in presence of spermidine this reversed the effect of spermidine and restored functional activity of NK cells, which was evident by increased expression of perforin and IFN- γ (Fig. 4). Similarly, a significantly increased expression of perforin and IFN- γ in NK cells incubated with DFMO plus Rosuvastatin was observed. Thus, this experiment suggests that DFMO plus Rosuvastatin combination has the ability to reverse polyamine inhibitory effects and can potentiate NK cell function (Fig. 4).

### DFMO and Rosuvastatin combination inhibits proliferation and induces cell death in colon tumors

**Figure 5:**
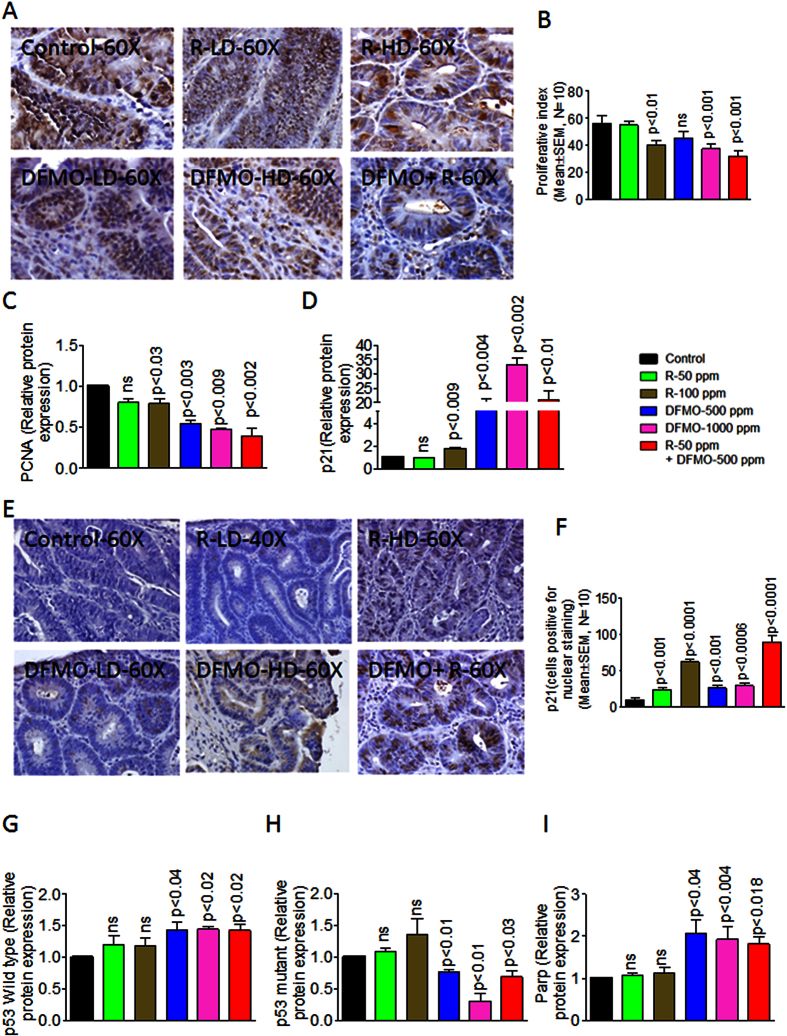
Immunohistochemical analysis of proliferation and apoptotic markers (**A**) Immunohistochemical staining for PCNA in colon tumors from rats fed control diet or fed with DFMO, Rosuvastatin or the combination of DFMO and Rosuvastatin. (**B**) quantification of proliferation in response to the treatments in A. A significant difference was observed in proliferative index between combination-treated and control group tumors. (**C**) A significant decrease was observed in PCNA protein expression between combination-treated and control group tumors. (**D**) A significant Increase was observed in p21 protein expression in combination-treated tumors compared to control group tumors. (**E**) Immunohistochemical staining for p21 in colon tumors from rats fed control diet or fed with DFMO, Rosuvastatin or the combination of DFMO and Rosuvastatin. (**F**) A significant Increase was observed in p21 nuclear expression in combination-treated tumors compared to control group tumors. (**G**) A significant Increase was observed in wild type p53 protein expression in combination-treated tumors compared to control group tumors. (**H**) A significant decrease was observed in mutant type p53 protein expression in combination-treated tumors compared to control group tumors. (**I**) A significant Increase was observed in PARP protein expression in combination-treated tumors compared to control group tumors.

DFMO and Rosuvastatin low dose combination reduced PCNA a proliferation marker in treated colon tumors compared to untreated control colon tumors (Fig. 5A). A non-significant decrease in PCNA with individual low doses was observed in colon tumors of animals fed DFMO and Rosuvastatin, compared to the colon tumors of control fed animals (Fig. 5A,B). The quantification of PCNA staining showed 56.2 ± 5.744 (mean ± SEM) PCNA-positive cells in control tumors, as compared with 54.80 ± 3.17, 39.80 ± 3.44, 45.40 ± 4.64, 37.20 ± 4.13, 32.00 ± 3.57 (mean ± SEM) PCNA positive cells in tumors from LD and HD Rosuvastatin-, LD and HD DFMO- and their low-dose combination–treated rats, respectively, accounting for a decrease in the proliferation index of about 43% (P < 0.001) with the combination treatment (Fig. 5B). Overall, PCNA protein expression in Rosuvastatin and DFMO treatment groups and low dose combination groups showed a significant decrease compared to untreated colon tumors (Fig. 1C, p < 0.002). DFMO treatment resulted in a dose-dependent increase of p21 protein expression in colon tumors (Fig. 5D). Rosuvastatin leads to an increase in p21 protein expression in colon tumors of high dose treatments. Whereas, low dose combination of DFMO and Rosuvastatin resulted in an increase of p21 protein expression in treated colon tumors compared to low dose individual doses (Fig. 5D). Immuno-histochemical analysis for p21 staining pattern revealed its increased nuclear expression by Rosuvastatin compared to DFMO (Fig. 3E). When combined together these agents induced high nuclear expression of p21 in low dose combination treatment in colon tumor cells (89.80 ± 8.072; p < 0.0001; Fig. 5F). DFMO treatment showed a dose-dependent increase in protein expression of wild type p53 expression (Fig. 5G), with a significant similar decrease in mutant p53 protein expression in colon tumors (Fig. 5H). Rosuvastatin treatment showed an increase in mutant p53 but failed to induce a significant wild type p53 protein expression in colon tumors (Fig. 5G,H). The combination of DFMO and Rosuvastatin resulted in a significant increase in wild type p53 protein expression (Fig. 5G,H). DFMO alone treatment showed significantly increased PARP protein expression and Rosuvastatin alone did not have any effect on PARP protein expression (Fig. 5I). Whereas, the combination treatments in colon tumors increased PARP protein expression compared to control tumors (Fig. 5I).

### DFMO and Rosuvastatin combination modulated cell proliferation signaling markers in colon tumors

**Figure 6:**
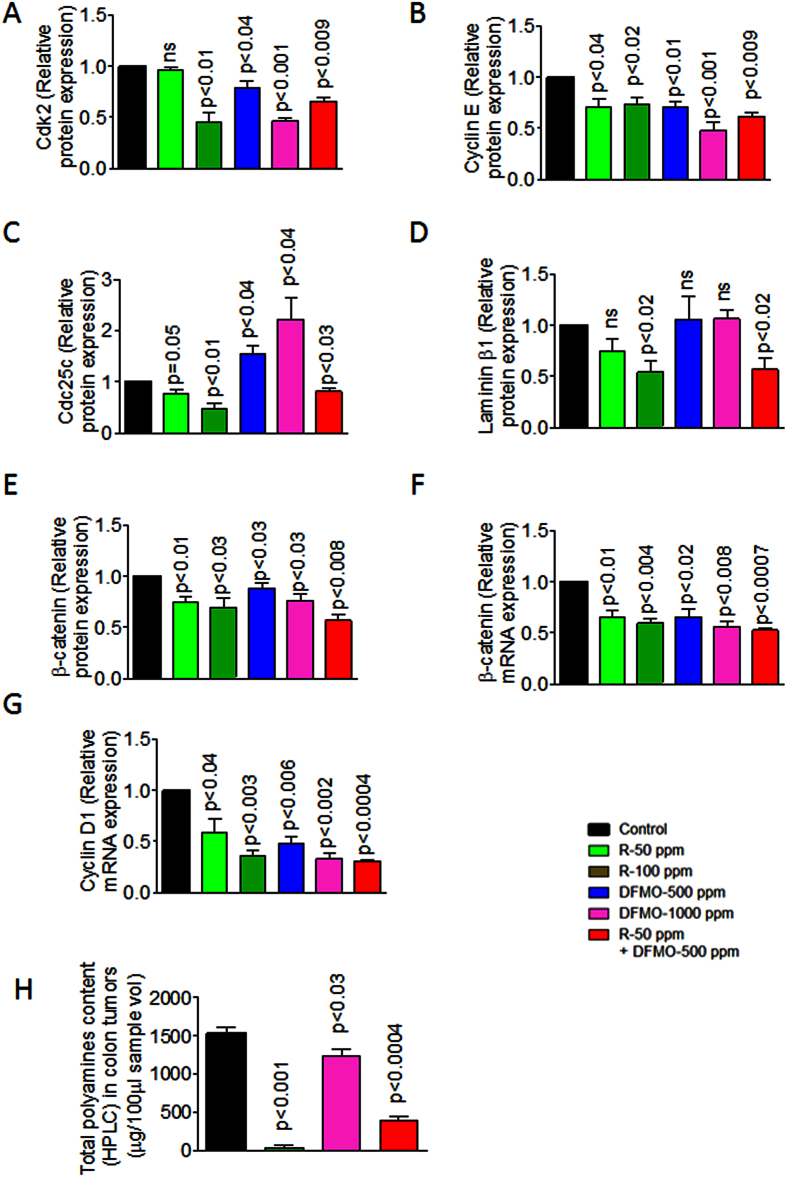
(**A**) A significant decrease was observed in Cdk2 protein expression in combination-treated tumors compared to control group tumors. (**B**) A significant decrease was observed in Cyclin E protein expression in combination-treated tumors compared to control group tumors. (**C**) A significant decrease was observed in Cdc25C protein expression in combination-treated tumors compared to control group tumors. (**D**) A significant decrease was observed in Laminin β1 protein expression in combination-treated tumors compared to control group tumors. (**E**) A significant decrease was observed in β-catenin protein expression in combination-treated tumors compared to control group tumors. (**F**) A significant decrease was observed in β-catenin mRNA expression in combination-treated tumors compared to control group tumors. (**G**) A significant decrease was observed in cyclin D1 mRNA expression in combination-treated tumors compared to control group tumors (**H**) A significant decrease was observed in Total polyamine content by fluorescent HPLC in combination-treated tumors compared to control group tumors.

DFMO and Rosuvastatin reduced protein expressions of cell cycle marker Cdk2 dose-dependently in colon tumors compared to untreated colon tumors. The combination of DFMO and Rosuvastatin showed a significant reduction in protein expressions of Cdk2 compared to individual low doses alone (Fig. 6A). High dose Rosuvastatin and both the doses of DFMO have a significant inhibitory effect on Cyclin E protein expression compared to untreated colon tumors (Fig. 6B). The low dose combination of DFMO and Rosuvastatin showed reduced expression of Cyclin E compared to individual low doses of these agents alone and control colon tumors (Fig. 6B). Rosuvastatin decreased Cdc25c protein expression in colon tumors, whereas DFMO resulted in increasing Cdc25c protein expression and the combination of DFMO and Rosuvastatin increased Cdc25c protein expression compared to control colon tumors (Fig. 6C). Rosuvastatin inhibited laminin 1β protein expression dose-dependently whereas DFMO did not show any effect on laminin 1β (Fig. 6D). However, the DFMO and Rosuvastatin combination in treated colon tumors showed decreased laminin 1β protein expression compared to control tumors (Fig. 6D).

DFMO, Rosuvastatin and combinations showed significant inhibition of β-catenin protein and mRNA expression compared to control and individual doses of each agent (Fig. 4E,F). A similar result was observed with cyclind1 mRNA expression which is downstream of β-catenin signaling pathway (Fig. 6G). Also, a significant decrease in cyclin d1 mRNA was observed in colon tumors of combination-dose treatment (Fig. 6G).

### DFMO, Rosuvastatin and its combination decreased polyamine content in colon tumors

Polyamine levels were measured via fluorescent HPLC system in control Vs treated colon tumors. As expected, DFMO was more effective in decreasing polyamine in colon tumors compared to Rosuvastatin alone treated colon tumors. DFMO and Rosuvastatin low dose combination was more effective in decreasing polyamines (~75%, Fig. 6H).

## Discussion

High amounts of polyamines have been consistently reported in colon carcinogenesis^25^. Elevated amounts of ODC in rectal mucosa are often associated with a high risk for CRC and it is suggested to be a potential biochemical marker of proliferation for CRC^26^,^27^,^28^,^29^. In the present study we have observed significant inhibition of adenomas, adenocarcinoma incidence and multiplicity in rat AOM model. No adenomas were noted in high dose DFMO treatments compared to control, suggesting DFMO’s role in inhibiting cell proliferation. A dose-dependent inhibition in colon adenocarcinoma incidence and multiplicity with increasing levels of DFMO was reported by *Reddy et al.*^30^. The present study results reiterate previously published results with DFMO^31^,^32^,^33^,^34^. Exogenous polyamines from dietary sources also add to the endogenous polyamines and the inhibition of both is important to completely block the effect of polyamines on tumor growth. Rosuvastatin was reported to inhibit arginase enzyme activity and ornithine levels, precursors of polyamines, and also polyamine levels during breast cancer development^35^. Also, Rosuvastatin shows effect on cholesterol pathway, affecting Kras/G protein transport mechanism resulting in limiting the entry of exogenous polyamines during colon tumor development. Hence, DFMO was combined with statins in small quantities to inhibit CRC progression in rat AOM model.

A very recent population-based cohort study reported reduced overall colorectal cancer mortality to 29% and increased survival in statin users^18^. Statins such as lovastatin, atorvastatin and pitavastatin have been effective in reducing AOM-induced neoplasia in rodents and in Apc min mice^10^,^11^,^12^,^13^. Rosuvastatin is more effective in reducing LDL cholesterol compared to other statins and our study helps in choosing the effective statin for CRC prevention trails. In the present study long-term dietary treatment with Rosuvastatin, i.e. 40 weeks after carcinogen administration in rats, significantly reduced colon adenocarcinoma multiplicity and incidence. Rosuvastatin did not display significant preventive effect on non-invasive adenocarcinoma multiplicity and incidence compared to DFMO alone and control diet fed animals. Whereas, Rosuvastatin was effective in reducing invasive adenocarcinoma multiplicity compared to control, suggesting its role in inhibiting the progression of non-invasive to invasive tumors. This may be due to the anti-proliferative effects of Rosuvastatin on the epithelial cells inhibiting the invasive capacity of the malignant cells. High-dose Rosuvastatin suppressed only invasive and total adenocarcinoma incidence (data not shown). This is the first study to show the chemopreventive effects of Rosuvastatin on CRC formation. The results of the Rosuvastatin study are further evidence for the potential of statins as a chemopreventive agent in colon carcinogenesis. A randomized trial for cancer prevention or therapy is needed to demonstrate similar effects by statins as this experimental evidence suggests the possible biological role of statins in inhibiting CRC.

The results of the present study indicate that when compared to individual doses of DFMO and Rosuvastatin, combinations of DFMO and Rosuvastatin induce greater inhibition of colon carcinogenesis. DFMO and Rosuvastatin combinations led to a significant decrease in both adenocarcinomas multiplicity and incidence, but a striking significant increase in adenomas were also observed. These results suggest that overall low dose combination treatments delayed the progression of adenomas to adenocarcinomas. Observations made in this study are particularly important because this can pave the way for the use of a combination of these agents in lower non-toxic doses whose added chemopreventive effect would be significant. As expected DFMO resulted in an increase of p21 and wild type p53 with a decrease in mutant p53, and increase in PARP which might have helped in the complete inhibition of adenomas in high dose DFMO. The combination of DFMO and Rosuvastatin did not show a similar effect on p21, wild type p53 and mutant p53. The combination of DFMO and Rosuvastatin had a significant inhibition of β-catenin and its downstream molecule cyclin D1 compared to control. There is substantial evidence that a large number of human CRCs and experimentally induced CRCs in animals contain a significant amount of β-catenin and cyclin d1^36^,^37^.

The mechanisms responsible for the inhibition of colon carcinogenesis by the DFMO and Rosuvastatin combination have not been fully established, it would appear from the various correlative marker analyses that the effect of these compounds in combination might be mediated through the cumulative effect of the individual inhibitory effects of these agents on various biomarkers of signaling, cell proliferation, cell cycle, and apoptosis. Increased polyamine uptake by immune cells also results in reduced cytokine production needed for anti-tumor activities and in anti-tumor immunity such as lymphokine-activated killer activities and a similar phenomenon was observed in other diseases^38^,^39^,^40^,^41^. Polyamine biosynthesis also plays a role in the intracellular regulation of interleukin 2 production which enhances cytotoxic effects of NK cells^42^. Importantly, these drugs alone or in combinations enhanced NK cells and also increased their ability to express perforin and IFN-γ. NK cells express diverse families of receptors which, upon activation, can trigger lysis of the infected or transformed cells^43^. Among them, NK1.1 and NKG2D are believed to play an important role in NK cell-mediated tumor cell recognition and cytolysis. Interestingly, the expression of NK1.1 in colon tumors is enhanced by DFMO and Rosuvastatin treatment. Increased NK cells are indicators of prognosis in cancer patients and most of the reports suggest progressive functional defects in NK cell proliferation and cytolytic activity in cancer patients. DFMO and Rosuvastatin have increased the proliferation of NK cells or might have increased the expression of NK1.1 receptor expressions on NK cells and increased NK1.1 positive NK cells with perforin and IFN-γ expressions which are released upon stimulation and necessary to lyse the target cell. Polyamines are reported to induce tumor protective immune suppression activities. Polyamine deprivation is reported to restore immune function by increasing NK cytotoxicity and to inhibit lung tumor formation and metastases in mice^44^,^45^. Our study showed that deprivation of polyamines by the combination of DFMO and Rosuvastatin inhibited tumor-induced immune suppression where a significant increase in intratumoral NK cells and also in perforin and IFN-γ expressions in NK cells was observed in treated colon tumors compared to untreated colon tumors.

In conclusion, the present study demonstrated that two dose levels of DFMO and Rosuvastatin when fed individually inhibited colon adenocarcinomas in a dose-dependent manner. In addition, the data herein reported indicates that the administration of DFMO and Rosuvastatin together in the diet was more effective in depriving polyamines and potentiating NKs than when administered individually and in suppressing the development of colon adenocarcinomas. The results of this study will help in developing these low dose combinations for tertiary prevention for recurrent malignant or premalignant lesions among resected CRC patients and will make a major contribution to the field of chemoprevention.

## Methods

### Reagents and antibodies

DFMO and Rosuvastatin were kindly provided by the Division of Cancer Prevention (DCP) Repository at the National Cancer Institute (Rockville, MD). Antibodies to detect mutant and wild type p53 were obtained from Abcam. Antibodies against p21, parp, laminin 1β, cdk2, cdc25c, cyclin E, perforin-1, IFN-γ and tubulin were from Santa Cruz Biotechnology. Perforin-1 and IFN-γ were conjugated using Apcc-Cy7 (Abcam) and PE-Cy5 (Novus Biological) conjugation kits respectively. Anti-mouse NK1.1-Apc (Clone PK136), anti-rat NK1.1 (clone 3.2.3) and respective isotype controls were from Biolegend. Anti-rat NGKG2D-PE (Clone 11D5F4) and isotype control were from e-Bioscience. Anti-rat Perforin and anti-rat IFN-γ were conjugated with fluorochrome Apc-Cy7 and PE-Cy5 respectively as per manufacturer’s instructions from Abcam. Antibodies for perforin, IFN-γ (SC-9105, SC-59992), isotype controls (Sc-3875, SC-2878) were from Santacruz Biotechnology. The carcinogen used to induce colon tumors in the current study was AOM, which was procured from Midwest Research Institute (Kansas MO).

### Animals and Experimental diets

Five week-old Male F344 rats were purchased from Harlan Breeding Laboratories (USA). Male F344 rats were quarantined for 10 days and had access to modified AIN-76A control diet^46^. Following quarantine, all animals were randomized by weight into various groups and transferred to an animal holding room. They were housed in plastic cages with filter tops under controlled conditions of a 12-h light/l2-h dark cycle, 50% humidity, and 21 °C temperature. All ingredients of the semi-purifed modified AIN-76A powdered diet were obtained from Bioserv (Flemington, NJ) and were stored at 4 °C prior to the preparation of diets. The experimental diets were prepared in our laboratory by adding a test agent (DFMO, Rosuvastatin or DFMO plus Rosuvastatin) to the control diet. The specific test agent was initially premixed with casein thoroughly by grinding. The incorporation of premix of various levels of test agents individually or in combination into the control diet was done with a Hobart mixer to ensure their uniform distribution. All control and experimental diets were prepared biweekly in our laboratory and stored in a cold room. Stability and uniformity of the diets were determined periodically, collected from top, middle and bottom of the diet storage bags. Animals had access to food and water at all times, and food cups were replenished with fresh diet twice weekly.

### Bioassay: Chemopreventive efficacy evaluation of agents individually and in combination

All animal experiments were performed in accordance with the institutional guidelines of the American Council on Animal Care and were approved by the Institutional Animal Care and Use Committee at the University of Oklahoma Health Sciences Center (OUHSC). At seven weeks of age Male F344 rats [36/group; 30 AOM treated, 6 vehicle (saline) treated] on AIN76A control diet, were injected with AOM (15 mg/Kg body weight)/saline *s.c*., once a week for two weeks (Fig. 1A). Eight weeks after AOM treatment rats were fed with experimental diets containing DFMO (500 and 1000 ppm), Rosuvastatin (50 and 100 ppm) and the combination of 500 DFMO and 50 Rosuvastatin. The test agents doses calculated were approximate clinically relevant doses. Animals were maintained on control or experimental diets until the termination of the experiment. Body weights were recorded every 2 weeks for the first 10 weeks and then every 4 weeks. Animals were monitored daily for general health. The experiment was terminated 49 weeks after the second AOM treatment, at which time all animals were sacrificed by CO_2_ euthanasia. After laparotomy, the entire small intestine, and large intestine were resected and opened longitudinally, and the contents were flushed with normal saline. Using a dissection microscope, colon and small intestinal tumors, if any, were noted grossly for their location and number.

### Histopathology and Immunohistochemistry

For histopathological evaluation of tumors, intestines were fixed in 10% neutral buffered formalin, processed and embedded in paraffin blocks, cut into multiple sections, and placed on slides for H&E staining. The slides stained with standard H&E protocol were examined by a pathologist, who was blinded to the treatment groups. The histological criteria used for the classification of intestinal tumors were as described previously^46^. The end points used were incidence and multiplicity of tumors where applicable. At the end of the study more than 96% of the colon tumors developed into adenocarcinomas in control diet fed animals. All adenocarcinomas were classified as invasive and non-invasive (Fig. 1B). The invasive adenocarcinomas were invading the muscularis mucosa deep into the intestinal wall and beyond. The noninvasive adenocarcinomas were those growing outward toward the intestinal lumen and not invading the muscularis mucosa. They were usually well-differentiated adenocarcinomas. The expression of protein markers was studied in control and treated colon tumor sample sections as described previously. After processing, the sections were incubated with primary antibodies to PCNA (1:1,500), p21 (1:500), Cav1 (1:500), and COX-2 (1:500), and then with appropriate secondary antibodies. Images of stained sections were captured with a bright-field microscope (Olympus AX71) connected to a digital imaging system with SPOT RT software version 3.0. Scoring was performed by two investigators blinded to the identity of the samples for PCNA-positive cells in the tumors (light microscopy at 400 X magnification). Tumor Cells with a brown nucleus were considered positive. The proliferation index was determined by dividing the number of positive cells by the total cells and multiplying by 100.

### Sample preparation and Derivatization for HPLC fluorescence detection

Colon tumors were homogenized and protein was measured by previously published method^47^. Deproteinization was then performed by mixing equal volumes of homogenate and 10% trichloroacetic acid. After vortex mixing and centrifugation at 14,800 g at 4 °C for 30 min, the supernatant was separated and the derivatization was performed in aliquots containing 100 g protein. 100 μl of bicarbonate/carbonate buffer (pH 8.5) and 500 μl 5.8 mM FMOC in HPLC-grade acetonitrile was added to 400 μl polyamine-containing sample. The mixture was gently swirled for 30 seconds prior to injection. Separation of the polyamine-FMOC derivatives was achieved with an aqueous (pH 6.1)/acetonitrile gradient of 60% to 100% in 20 min at a flow rate of 1.0 mL min^−1^. The wavelength of excitation was set at 265 nm and fluorescence emission was monitored through 340 nm (Shimadzu RF535).

### Tumor dissociation and Flow cytometry

At the termination of the experiment, rat colon tumors were dissociated for isolation of NK cells using tumor dissociation kits, a gentleMACS Dissociator and a MACSmixTM Tube Rotator according to manufacturer’s instructions (Miltenyi Biotech, CA). The NK cells and NK cells expressing perforin/IFN-γ were determined in dissociated tumor samples using antibodies to NK1.1, NKG2D, perforin, and IFN-γ. The single cell suspensions were stained as reported previously by us^48^ and was run on a Stratedigm S1200Ex flow cytometer and data was acquired using the CellCapTure acquisition and analysis software.

### Isolation of NK cells and *ex-vivo* analysis of DFMO, Rosuvastatin and its combination effects on NK cells

Splenic untouched NK cells from 6–7 week-old C57BL/6 J mice were isolated using NK cell isolation Kit II, mouse (Miltenyi). Isolated NK cells were equally dispensed in various wells and exposed to DFMO (18 μg), Rosuvastatin (24 μg) and combinations in presence of polyamine, spermidine (1 μg) for 30 min at 37 °C and stained and run on FACSCalibur as described above to analyze the effects of polyamine and drugs on NK cell phenotype and expressions of IFN-γ and perforin by NK cells.

### Protein Expression by western Immunoblotting

Colon tumors exposed to various concentrations of DFMO, Rosuvastatin and combinations snap frozen were slowly thawed and processed for protein and estimated protein content using the Bio-Rad Protein Assay reagent as mentioned previously^46^. After running SDS-PAGE with equal concentrations of protein, they were transferred onto nitro-cellulose membranes. After blocking in 5% milk, the membranes were incubated with primary antibodies to PCNA, p21, laminin 1β, Cyclin E, Cyclin D1, Cdk2, Cdc25c, β-catenin, mutant p53, wild type p53, parp (1:500, in TBS Tween 20 solution), then probed with horseradish peroxidase–conjugated secondary antibody. The Super Signal West Pico Chemiluminescence procedure developed by Pierce Detection was used for developing the membranes. The bands were captured on Ewen Parker Blue sensitive X-ray films. Later the protein bands were analyzed by densitometry using image quant software. Tubulin antibody was used to confirm equal protein loading.

### mRNA expressions by Reverse transcription-PCR

Total RNA from tumor samples was extracted using TRIzol (Life Technology) and transformed to complementary DNA (Life Technology) as per the manufacturer’s instructions. PCR was carried out as described previously using Taq polymerase Master Mix (Phenix) (46). PCR primers and conditions for β-catenin, and cyclin D1 are mentioned in Table 1. The PCR bands were visualized and captured under UV illumination.

### Statistical Analysis

Tumor multiplicity (total number of colon tumors per rat) was calculated for each group, and the significance of the differences between the control diet and experimental diets was analyzed using the unpaired Student test, with Welch’s correction. The colon tumor incidence (total number of colon tumor-bearing rats with respect to the total number of rats at risk) between the animals fed the control diet and experimental diets was analyzed using “Fishers exact test”. Differences were considered statistically significant at P < 0.05. All statistical analysis was conducted using GraphPad Prism Software 6.0 (GraphPad Software, Inc.).

## Additional Information

**How to cite this article**: Janakiram, N. B. *et al.* Potentiating NK cell activity by combination of Rosuvastatin and Difluoromethylornithine for effective chemopreventive efficacy against Colon Cancer. *Sci. Rep.* **6**, 37046; doi: 10.1038/srep37046 (2016).

**Publisher's note:** Springer Nature remains neutral with regard to jurisdictional claims in published maps and institutional affiliations.

## Figures and Tables

**Table 1:** The oligonucleotide primer sequences and PCR conditions for β-catenin and cyclin D1.

Gene	Primer sequence	PCR condition
β-catenin	5°-CGGGATCCACAAGAAACGGCTTTCA-3° (sense) and 5°-GAGAATTCCAGGTCAGTATCAAACCA-3° (antisense).	94 °C for 3 minutes, followed by 35 cycles at 94 °C for 30 seconds, 55 °C for 1 minute, and 72 °C for 1 minute.
Cyclin D1	5°-CTGGCCATGAACTACCTGGA-3° (sense) and 5°-GTCACACTTGATCACTCTGG-3° (antisense).	94 °C for 3 minutes, followed by 35 cycles at 94 °C for 30 seconds, 60 °C for 20 seconds, and 72 °C for 45 seconds.
